# A Novel Presentation of Euglycemic Diabetic Ketoacidosis Associated with SGLT2 Inhibitor and Weekly GLP-1 Agonist: Case Report

**DOI:** 10.3390/healthcare13172245

**Published:** 2025-09-08

**Authors:** Young Sang Lyu

**Affiliations:** Department of Endocrinology and Metabolism, Chosun University Hospital, School of Medicine, Chosun University, Gwangju 61452, Republic of Korea; lyu0923@naver.com

**Keywords:** euglycemic diabetic ketoacidosis, SGLT2 inhibitor, GLP-1 receptor agonist

## Abstract

**Background:** Sodium–glucose cotransporter-2 (SGLT2) inhibitors are widely used to manage type 2 diabetes mellitus (T2DM) because of their glucose-lowering and cardioprotective effects. However, euglycemic diabetic ketoacidosis (euDKA) is an uncommon but serious adverse event. EuDKA is characterized by metabolic acidosis and ketosis with only mild-to-moderate hyperglycemia, making diagnosis challenging. The risk of this interaction may be increased with the concurrent use of glucagon-like peptide-1 receptor agonists (GLP-1RAs), particularly during periods of reduced caloric intake or the presence of gastrointestinal symptoms. **Case:** A 38-year-old woman with newly diagnosed T2DM presented with five days of fatigue, poor oral intake, nausea, and vomiting. She had recently initiated semaglutide (GLP-1RA) for weight loss and practiced prolonged intermittent fasting. One week prior, she had started metformin and enavogliflozin, a selective SGLT2 inhibitor. Laboratory results showed a glucose level of 137 mg/dL, urine ketones (+++), lactate level of 4.87 mg/dL, HbA1c of 9.3%, C-peptide of 0.88 ng/mL, and high anion gap metabolic acidosis. She was diagnosed with euDKA and treated with IV fluids, insulin infusion, dextrose, and potassium supplementation. Her symptoms resolved, and she was discharged in a stable condition. **Conclusion:** This case highlights the importance of recognizing euDKA in patients using SGLT2 inhibitors and GLP-1RAs, particularly those with fasting or gastrointestinal symptoms. Clinicians should suspect euDKA even without significant hyperglycemia to enable prompt diagnosis and management, thereby preventing complications.

## 1. Introduction

Sodium–glucose cotransporter-2 (SGLT2) inhibitors have emerged as a cornerstone in the management of type 2 diabetes mellitus (T2DM). They offer significant benefits beyond glycemic control, including reductions in body weight and improvements in cardiovascular and renal outcomes [1,2,3,4]. Consequently, their use has rapidly expanded in clinical practice for both glycemic control and cardiometabolic risk reduction. Despite these advantages, SGLT2 inhibitors are associated with a distinct spectrum of adverse metabolic effects, among which euglycemic diabetic ketoacidosis (euDKA) is of particular concern [5,6,7].

EuDKA is a rare but serious complication of diabetes, characterized by a triad of high anion gap metabolic acidosis, ketonemia, and mild-to-moderate hyperglycemia, often defined as a serum glucose level < 250 mg/dL [8]. This atypical biochemical presentation can delay diagnosis and treatment, especially in patients with nonspecific symptoms, such as nausea, vomiting, or fatigue. The pathogenesis of SGLT2 inhibitor–associated DKA involves multiple mechanisms, including reduced insulin secretion due to glycosuria-induced normoglycemia, elevated glucagon levels, enhanced lipolysis, and increased hepatic ketogenesis [7,9,10,11]. In contrast to classic DKA, euDKA lacks pronounced hyperglycemia, which often prompts urgent evaluation and treatment, making clinical suspicion critical.

Furthermore, the clinical picture becomes more complex with the increasing use of combination therapies, particularly glucagon-like peptide-1 receptor agonists (GLP-1RAs), such as semaglutide. These agents are beneficial for weight loss and glycemic control but frequently cause gastrointestinal side effects, such as anorexia, nausea, and vomiting. This can lead to reduced caloric intake, dehydration, and catabolic stress, all of which are recognized precipitating factors for DKA [5,12]. In addition, GLP-1RAs delay gastric emptying and suppress insulin secretion, further contributing to the ketotic milieu in susceptible individuals.

Recent reports have highlighted an increased risk of euDKA with the concurrent use of SGLT2 inhibitors and GLP-1RAs, especially in patients on low-calorie diets, intermittent fasting, or experiencing periods of physiological stress [12,13]. However, awareness of this risk is limited. The early symptoms of DKA may be misattributed to the known side effects of GLP-1RA therapy, potentially resulting in a delay in diagnosis.

In this report, we describe a case of euDKA in a previously healthy woman with newly diagnosed T2DM who developed severe metabolic acidosis shortly after initiating semaglutide and enavogliflozin treatment with prolonged intermittent fasting. This case underscores the importance of clinical awareness, timely recognition of atypical DKA presentations, and careful patient education when prescribing these agents. This case report was prepared in accordance with the CARE (CAse REport) guidelines to ensure methodological transparency and completeness.

## 2. Case Presentation

A 38-year-old woman (height, 165 cm; weight, 70 kg; BMI, 25.7 kg/m^2^) presented to our tertiary hospital with a 5-day history of progressive fatigue and decreased appetite. Apart from a recent diagnosis of diabetes, she had no significant medical history, prior surgeries, or regular medication use. She presented to the emergency department with generalized weakness, nausea, and vomiting. There was no family history of diabetes or endocrine disorders, and no history of smoking, alcohol consumption, or illicit drug use. Three weeks before presentation, she was started on subcutaneous semaglutide (0.25 mg weekly) for weight loss. One week before admission, she was diagnosed with T2DM at a local clinic and was prescribed metformin 1000 mg/day and enavogliflozin 0.3 mg/day.

The patient had also been practicing intermittent fasting since starting semaglutide, following a 20:4 dietary pattern (20 h fasting and 4-h eating window) to enhance weight loss. In addition, she adopted a low-carbohydrate, high-protein diet during the eating window, which may have further restricted carbohydrate intake and promoted ketone body production. Since starting antidiabetic medications, she reported persistent nausea and intermittent vomiting, which she attributed to the common side effects of semaglutide. Therefore, she did not seek immediate medical care. She had no history of ketonuria or ketoacidosis. Although she experienced a modest reduction in appetite and mild weight loss after starting semaglutide, there was no history of unintentional or progressive weight loss before initiating antidiabetic therapy. As her symptoms worsened, she was admitted to a local hospital four days before transfer for suspected euDKA. Despite treatment, the acidosis did not improve, and she was referred to our institution.

Initial laboratory evaluation at the local clinic revealed a fasting glucose level of 137 mg/dL, strongly positive urine ketones (+++), normal serum lactate (4.87 mg/dL; reference range, 4.5–19.8), HbA1c of 9.3%, and C-peptide level of 0.88 ng/mL (Table 1). Arterial blood gas analysis at our hospital demonstrated high anion gap metabolic acidosis (pH 7.269, pCO_2_ 25.3, pO_2_ 106, HCO_3_ 11.6, and anion gap 17), which was consistent with diabetic ketoacidosis. She was afebrile, had no leukocytosis or localizing signs, and her CRP level was within the normal range, making infection an unlikely precipitating factor. Considering the relatively low serum glucose levels and biochemical findings, she was diagnosed with euDKA.

### 2.1. Treatment and Progress

Upon admission, the patient was moderately to severely dehydrated. Intravenous fluid resuscitation was promptly initiated using a balanced crystalloid solution at 160 mL/h. Simultaneously, a standard treatment protocol for diabetic ketoacidosis (DKA) was implemented. To suppress ketogenesis and gradually lower circulating ketone levels, a continuous intravenous insulin infusion was started at 1–2 units/h. Because of the patient’s relatively low serum glucose level (137 mg/dL), an 18% dextrose solution was co-administered at 40 mL/h to prevent hypoglycemia. Electrolyte correction was performed with potassium chloride (KCl) at a concentration of 40 mEq/L and an infusion rate of 80 mL/h to prevent hypokalemia during insulin therapy. Electrolytes were closely monitored and adjusted as required. On admission, tests for type 1 diabetes–related antibodies, including GAD and islet cell antibodies, were negative, suggesting a low likelihood of autoimmune diabetes, such as LADA.

Within 48 h, both the patient’s clinical condition and laboratory findings improved (Table 1). Nausea and vomiting subsided, and the patient tolerated oral fluid intake. By hospital day 3, the anion gap had decreased significantly, and serum bicarbonate levels had begun to rise, suggesting resolution of metabolic acidosis. Consequently, the insulin infusion rate was gradually reduced. Once the anion gap was normalized, intravenous insulin was transitioned to subcutaneous insulin. A once-daily basal insulin regimen was initiated to maintain glycemic control and prevent recurrence of ketogenesis. Both the SGLT2 inhibitor and the GLP-1 receptor agonist, suspected to be contributing factors to the development of euDKA, were discontinued.

Throughout hospitalization, blood glucose levels and arterial blood gas analyses were closely monitored to assess response to therapy. Dextrose infusion rates were adjusted to maintain blood glucose levels within the target range of 140–180 mg/dL, ensuring continuous insulin activity against ketone production. The patient’s mental status, dietary intake, and hemodynamic stability improved steadily. By hospital day 5, her appetite had returned, she tolerated a regular diet without nausea, and her serum chemistry values had stabilized. On day 6, her high anion gap metabolic acidosis had fully resolved, urinary ketones had cleared, and she was discharged in a stable condition.

Before discharge, comprehensive education was provided to prevent the recurrence of DKA. Topics included sick-day management strategies, medication adherence, and the importance of maintaining adequate nutrition. The SGLT2 inhibitor, which was considered a precipitating factor for euDKA in this case, was permanently discontinued. GLP-1RA (semaglutide) was also withheld because of its gastrointestinal side effects, and the patient’s associated fasting behavior was suspected to be a contributing factor. Although a short-term basal–bolus regimen is often recommended following recovery from DKA, this approach was not feasible for our patient because of lifestyle constraints that prevented consistent insulin administration at mealtimes. Therefore, she was switched to a conservative regimen of basal insulin, metformin, and a DPP-4 inhibitor. The patient remained clinically stable and was discharged on hospital day 6.

### 2.2. Outpatient Follow-Up

At the two-week follow-up visit, the patient remained clinically stable, with no recurrence of ketosis. She adapted well to the basal insulin regimen, and her self-monitored blood glucose values were within the target range. Intermittent fasting was discontinued, and the patient resumed a normal diet. No gastrointestinal symptoms or abdominal discomfort were reported, and her overall health status improved. Semaglutide was not resumed because its gastrointestinal side effects, combined with prolonged fasting, were considered potential contributors to the development of euDKA. Instead, the patient was maintained on a conservative regimen comprising basal insulin, metformin, and a DPP-4 inhibitor. During three months of follow-up after discharge, she remained stable on this regimen, with good glycemic control and no further episodes of acidosis. A structured overview of the patient’s clinical course, including symptom onset, hospital management, and follow-up outcomes, is shown in Table 2.

## 3. Discussion

This case highlights a clinically significant episode of euDKA in a patient with newly diagnosed T2DM who was initiated on combination therapy with a GLP-1RA and an SGLT2 inhibitor. Although both drug classes are widely prescribed for their beneficial effects on glycemic control, weight reduction, and cardiometabolic outcomes, clinicians should be aware that they may increase the risk of ketone accumulation and ketoacidosis, particularly during physiological stress.

### 3.1. Pathophysiological Considerations

SGLT2 inhibitors have transformed the management of T2DM by promoting urinary glucose excretion and lowering plasma glucose levels, independent of insulin. In addition to improving glycemic control, they provide cardiovascular and renal protection, supporting their widespread clinical use [2,14,15]. However, their mechanisms of action are inherently linked to increased ketone production. By inducing glycosuria, SGLT2 inhibitors reduce circulating insulin levels, creating a relatively insulin-deficient environment. At the same time, they stimulate glucagon secretion and enhance lipolysis, increasing free fatty acid availability for hepatic ketogenesis [6,7,11]. When combined with precipitating factors such as reduced oral intake, dehydration, infection, or acute illness, this metabolic shift may be amplified, predisposing patients to DKA even in the absence of significant hyperglycemia [7,16,17]. Reports of euglycemic DKA following SGLT2 inhibitor therapy support these proposed mechanisms [18,19].

In contrast, GLP-1 receptor agonists may exacerbate ketone accumulation through gastrointestinal side effects. In our case, semaglutide was prescribed for weight reduction and induced progressive nausea, anorexia, and prolonged fasting. By delaying gastric emptying and suppressing appetite, GLP-1RAs can promote calorie restriction, further enhancing ketogenesis [13,20,21]. Therefore, the concomitant use of an SGLT2 inhibitor and GLP-1RA creates a synergistic risk of euDKA, particularly under conditions of caloric restriction or dehydration. The close temporal relationship between drug initiation and symptom onset, pharmacological plausibility, absence of alternative triggers, and prompt resolution after discontinuation strongly support a drug-associated mechanism. Although prolonged intermittent fasting may have contributed, the severity of acidosis, temporal association, and rapid recovery with insulin–dextrose therapy point toward medication-induced euDKA rather than pure starvation ketoacidosis.

The incidence of enavogliflozin-associated euDKA has not been systematically reported, and no large-scale comparative data are currently available. Enavogliflozin is a potent and selective SGLT2 inhibitor that has been shown to induce greater urinary glucose excretion than dapagliflozin at equivalent doses, and clinical studies suggest superior glucose-lowering efficacy even in the presence of mild renal impairment [22,23,24]. This pharmacological potency may theoretically predispose patients to enhanced ketogenesis and a higher risk of euDKA than other SGLT2 inhibitors. However, direct clinical evidence supporting the increased incidence of enavogliflozin-associated euDKA is lacking. Future studies are warranted to clarify whether the pharmacodynamic properties of enavogliflozin translate into a clinically meaningful difference in euDKA risk across the SGLT2 inhibitor class.

### 3.2. Unique Aspects of the Present Case

Unlike most previous case reports that only described “standard DKA management” [13,25], our report provides a detailed account of the therapeutic approaches. Specifically, we initiated continuous intravenous insulin infusion at 1–2 units per hour. This was co-administered with an 18% dextrose solution at 40 mL/h and potassium chloride at a concentration of 40 mEq/L, infused at 80 mL/h. This detailed description offers practical insights into the challenges of balancing insulin administration with the prevention of hypoglycemia and hypokalemia in patients with euDKA. Moreover, unlike previous reports that provided limited discussion on long-term prevention, our case places particular emphasis on structured patient education. At discharge, the educational topics included sick day management strategies, medication adherence, and the importance of maintaining adequate nutrition. The patient was also advised to discontinue prolonged intermittent fasting, which was considered a significant precipitating factor. This reinforcement of preventive measures contributed to favorable long-term outcomes, with no recurrence of ketoacidosis observed during the follow-up period.

### 3.3. Clinical Implications

The patient presented with severe fatigue, vomiting, and anorexia. Although the blood glucose level was 137 mg/dL, laboratory findings revealed high anion gap metabolic acidosis and strong ketonuria, confirming the diagnosis of euDKA [26]. Because severe hyperglycemia is absent in such cases, euDKA is often overlooked. Therefore, a high index of suspicion is warranted in patients receiving GLP-1RA or SGLT2i therapy who present with unexplained acidosis. Recent studies have suggested that euDKA may be associated with worse clinical outcomes than classical DKA, underscoring the importance of early recognition and management [8].

### 3.4. Therapeutic Considerations

The management of euDKA generally follows the standard protocols established for classical DKA, which include prompt intravenous fluid resuscitation, continuous insulin infusion, and correction of electrolyte imbalances, most notably, potassium levels. In this case, the application of these standard measures led to rapid resolution of metabolic acidosis and a favorable clinical outcome [25,26,27]. However, euDKA presents several unique therapeutic challenges compared to hyperglycemic DKA, with notable differences in insulin administration strategie. Since blood glucose levels in euDKA typically remain near normal or only mildly elevated (usually <250 mg/dL), a major clinical concern is determining the effective mode of insulin administration without inducing hypoglycemia. Insulin is essential for suppressing lipolysis, inhibiting hepatic ketogenesis, and facilitating the clearance of circulating ketone bodies.

However, in the absence of significant hyperglycemia, insulin therapy alone may cause profound hypoglycemia. To prevent this, dextrose-containing fluids, commonly 5–10% dextrose in normal saline or half-normal saline, are co-administered alongside insulin infusion. This approach allows for continuous insulin activity while maintaining appropriate blood glucose levels. This dual-therapy strategy (insulin–dextrose combination therapy) requires frequent monitoring of blood glucose and ketone levels (or anion gap) and serum electrolytes, particularly potassium, to enable careful titration of both insulin and dextrose infusion rates. Because insulin drives potassium intracellularly and promotes renal excretion, hypokalemia is a common and potentially life-threatening complication of treatment. As such, aggressive potassium supplementation is required to maintain serum potassium levels within the target range of 4.0–5.0 mEq/L and to prevent cardiac arrhythmias or neuromuscular complications.

### 3.5. Limitations

This study has several limitations. First, serum β-hydroxybutyrate levels were not measured due to laboratory constraints, and the diagnosis of euDKA was based on high anion gap metabolic acidosis, ketonuria, mild hyperglycemia, and the clinical context. Second, as this is a single case report, causal inference is limited; hence, the findings cannot be generalized to all patients receiving SGLT2 inhibitors or GLP-1 receptor agonists. Finally, dietary details were obtained retrospectively from patient self-reports, which may have been subject to recall bias. Despite these limitations, this case highlights a clinically important and potentially under-recognized adverse event. This also emphasizes the need for heightened vigilance and patient education when prescribing combinations of these agents.

## 4. Conclusions

SGLT2 inhibitors are beneficial for treating type 2 diabetes but can precipitate euglycemic DKA. To the best of our knowledge, this case is unique in representing the first reported episode of euDKA in a newly diagnosed patient with T2DM treated with a combination of semaglutide and enavogliflozin during prolonged intermittent fasting. Early recognition and aggressive management are crucial for achieving favorable outcomes. Before initiating SGLT2 inhibitors and GLP-1 receptor agonists, clinicians should carefully evaluate potential risk factors, such as fasting, dehydration, and gastrointestinal intolerance, and provide thorough patient education regarding the warning symptoms of DKA.

## Figures and Tables

**Table 1:** Serial changes in laboratory parameters during hospitalization.

Variable	0 h	+24 h	+48 h	+72 h	Normal Reference Range
Glucose (mg/dL)	131	134	137	139	70–100
HbA1c (%)	9.3				3.9–6.1
C-peptide (ng/mL)	0.88				0.48–3.30
Creatinine (mg/dL)	0.71	0.70	0.57	0.50	0.4–1.30
Sodium (mEq/L)	131	134	137	139	136–144
Potassium (mEq/L)	4.0	3.4	3.5	3.9	3.3–5.5
Chloride (mEq/L)	101	102	104	102	99–111
Arterial pH	7.269	7.282	7.321	7.336	7.35–7.45
pCO_2_ (mmHg)	25.3	24.7	30.2	34.2	35–45
pO_2_ (mmHg)	103	89	92	86	83–108
Bicarbonate (mEq/L)	11	16	9	27	17–29
Anion gap (mEq/L)	19	16	15	10	7–17
Total bilirubin (mg/dL)	1.4	1.0	1.2	1.2	≤1.2
AST (U/L)	34	30	20	24	10–35
ALT (U/L)	28	26	18	18	10–35
Lipase (U/L)	90	40	35	38	12–53
Lactic acid (mg/dL)	16	10	8	6	4.5–19.8
WBC count (/µL)	8269	7462	4151	5986	4000–10,800
CRP (mg/dL)	0.3	0.5	0.4		0–0.3

Abbreviations: ALT, alanine aminotransferase; AST, aspartate aminotransferase; CRP, C-reactive protein; WBC, white blood cell.

**Table 2:** Timeline of clinical events, interventions, and outcomes.

Date/Hospital Day	Clinical Events	Interventions	Outcomes
Day −14 to 0 (Outpatient)	Initiated semaglutide for weight reduction; began 20:4 intermittent fasting with a high-protein diet; developed progressive nausea and poor oral intake	–	Weight loss; prolonged fasting state
Day 0 (ER Visit)	Presented with weakness, nausea, and vomiting; blood glucose (BG): 137 mg/dL; positive urine ketones; high anion gap metabolic acidosis	Intravenous fluids, insulin infusion, electrolyte monitoring; SGLT2 inhibitor and GLP-1RA discontinued	Diagnosis: Euglycemic diabetic ketoacidosis (DKA)
Day 1–5 (Inpatient)	Persistent metabolic acidosis with gradual clinical improvement	Insulin–dextrose infusion, potassium replacement, supportive care	Resolution of acidosis; normalization of ketones
Day 6 (Discharge)	Clinically stable; gastrointestinal symptoms resolved	Initiated basal insulin + metformin + DPP-4 inhibitor; patient education provided	Discharged in stable condition
2-month follow-up	No recurrence of ketosis; regular diet resumed; intermittent fasting discontinued	Maintained on the same regimen	Good glycemic control; no acidosis

Abbreviations: DKA, diabetic ketoacidosis; GLP-1RA, glucagon-like peptide-1 receptor agonist.

## Data Availability

All data supporting the findings of this case are available from the corresponding author upon reasonable request.

## Abbreviations

The following abbreviations are used in this manuscript:

BMI	Body Mass Index
DKA	Diabetic Ketoacidosis
DPP-4	Dipeptidyl Peptidase-4
euDKA	Euglycemic Diabetic Ketoacidosis
GLP-1RA	Glucagon-Like Peptide-1 Receptor Agonist
SGLT2	Sodium-Glucose Cotransporter-2

## References

[1] Bell R.M., Yellon D.M. (2018). SGLT2 inhibitors: Hypotheses on the mechanism of cardiovascular protection. Lancet Diabetes Endocrinol..

[2] Wiviott S.D., Raz I., Bonaca M.P., Mosenzon O., Kato E.T., Cahn A., Silverman M.G., Zelniker T.A., Kuder J.F., Murphy S.A. (2019). Dapagliflozin and cardiovascular outcomes in type 2 diabetes. N. Engl. J. Med..

[3] Chan J.C., Chan M.C. (2023). SGLT2 inhibitors: The next blockbuster multifaceted drug?. Medicina.

[4] Andreea M.M., Surabhi S., Razvan-Ionut P., Lucia C., Camelia N., Emil T., Tiberiu N.I. (2023). Sodium-glucose cotransporter 2 (SGLT2) inhibitors: Harms or unexpected benefits?. Medicina.

[5] Modi A., Agrawal A., Morgan F. (2017). Euglycemic diabetic ketoacidosis: A review. Curr. Diabetes Rev..

[6] Chow E., Clement S., Garg R. (2023). Euglycemic diabetic ketoacidosis in the era of SGLT-2 inhibitors. BMJ Open Diabetes Res. Care.

[7] Ogawa W., Sakaguchi K. (2016). Euglycemic diabetic ketoacidosis induced by SGLT2 inhibitors: Possible mechanism and contributing factors. J. Diabetes Investig..

[8] Koceva A., Kravos Tramšek N.A. (2024). From sweet to sour: SGLT-2-inhibitor-induced euglycemic diabetic ketoacidosis. J. Pers. Med..

[9] Taylor S.I., Blau J.E., Rother K.I. (2015). SGLT2 inhibitors may predispose to ketoacidosis. J. Clin. Endocrinol. Metab..

[10] Sargent J. (2015). SGLT2 inhibitor dapagliflozin promotes glucagon secretion in α islet cells. Nat. Rev. Endocrinol..

[11] Saponaro C., Pattou F., Bonner C. (2018). SGLT2 inhibition and glucagon secretion in humans. Diabetes Metab..

[12] Butt H., Rajagopalan D., Ahsan S., Ahmad A., Wettimuny S., De La Torre A., Shah R. (2025). Clinical Challenge: Managing Euglycemic Diabetic Ketoacidosis in a Post-Whipple Procedure Patient on SGLT-2 Inhibitors and GLP-1 Agonist. Am. J. Respir. Crit. Care Med..

[13] Louwagie E.J., Diego J.N., Farooqi C.S., Kamal M.M. (2025). Euglycemic Ketoacidosis Following Coadministration of an SGLT2 Inhibitor and Tirzepatide. JCEM Case Rep..

[14] Heerspink H.J., Perkins B.A., Fitchett D.H., Husain M., Cherney D.Z. (2016). Sodium glucose cotransporter 2 inhibitors in the treatment of diabetes mellitus: Cardiovascular and kidney effects, potential mechanisms, and clinical applications. Circulation.

[15] Zinman B., Wanner C., Lachin J.M., Fitchett D., Bluhmki E., Hantel S., Mattheus M., Devins T., Johansen O.E., Woerle H.J. (2015). Empagliflozin, cardiovascular outcomes, and mortality in type 2 diabetes. N. Engl. J. Med..

[16] Selwyn J., Pichardo-Lowden A.R. (2023). Managing hospitalized patients taking SGLT2 inhibitors: Reducing the risk of euglycemic diabetic ketoacidosis. Diabetology.

[17] Morace C., Lorello G., Bellone F., Quartarone C., Ruggeri D., Giandalia A., Mandraffino G., Minutoli L., Squadrito G., Russo G.T. (2024). Ketoacidosis and SGLT2 inhibitors: A narrative review. Metabolites.

[18] Wang K.M., Isom R.T. (2020). SGLT2 inhibitor–induced euglycemic diabetic ketoacidosis: A case report. Kidney Med..

[19] Alkatheeri A., Alseddeeqi E. (2022). Euglycemic diabetic ketoacidosis induced by sodium-glucose cotransporter 2 inhibitor in the setting of prolonged fasting: A case report. J. Med. Case Rep..

[20] Filippatos T.D., Panagiotopoulou T.V., Elisaf M.S. (2015). Adverse effects of GLP-1 receptor agonists. Rev. Diabet. Stud. RDS.

[21] Sood N., Bansal O., Garg R., Hoskote A. (2024). Euglycemic Ketoacidosis From Semaglutide in a Patient Without Diabetes. JCEM Case Rep..

[22] Lyu Y.S., Hong S., Lee S.E., Cho B.Y., Park C.-Y. (2024). Efficacy and safety of enavogliflozin vs. dapagliflozin as add-on therapy in patients with type 2 diabetes mellitus based on renal function: A pooled analysis of two randomized controlled trials. Cardiovasc. Diabetol..

[23] Yang Y.S., Min K.W., Park S.O., Kim K.S., Yu J.M., Hong E.G., Cho S.R., Won K.C., Kim Y.H., Oh S. (2023). Efficacy and safety of monotherapy with enavogliflozin in Korean patients with type 2 diabetes mellitus: Results of a 12-week, multicentre, randomized, double-blind, placebo-controlled, phase 2 trial. Diabetes Obes. Metab..

[24] Choi M.-K., Nam S.J., Ji H.-Y., Park M.J., Choi J.-S., Song I.-S. (2020). Comparative pharmacokinetics and pharmacodynamics of a novel sodium-glucose cotransporter 2 inhibitor, DWP16001, with dapagliflozin and ipragliflozin. Pharmaceutics.

[25] Dhatariya K.K., Umpierrez G.E. (2017). Guidelines for management of diabetic ketoacidosis: Time to revise?. Lancet Diabetes Endocrinol..

[26] El-Remessy A.B. (2022). Diabetic ketoacidosis management: Updates and challenges for specific patient population. Endocrines.

[27] Munsakul N., Manosroi W., Buranapin S. (2024). Predictors and Predictive Score of In-Hospital Mortality in Diabetic Ketoacidosis: A Retrospective Cohort Study. Medicina.

